# Reciprocal Relations between Cognitive Empathy and Post-Traumatic Growth in School Bullying Victims

**DOI:** 10.3390/bs14060435

**Published:** 2024-05-23

**Authors:** Fang Liu, Bo Chen, Xinrong Liu, Yifan Zheng, Xiao Zhou, Rui Zhen

**Affiliations:** 1 Jing Hengyi School of Education, Hangzhou Normal University, Hangzhou 311121, China; 2 Department of Psychology and Behavioral Sciences, Zhejiang University, Hangzhou 310058, China; 3 Zhejiang Philosophy and Social Science Laboratory for Research in Early Development and Childcare, Hangzhou Normal University, Hangzhou 310030, China

**Keywords:** school bullying victimization, adolescents, cognitive empathy, post-traumatic growth, temporal relations

## Abstract

The association between post-traumatic growth (PTG) and cognitive empathy is well documented; however, few studies have tested the causal pathways explaining this association in school bullying victims’ later recovery and growth in the long term. This study used a longitudinal design to examine the reciprocal relations between cognitive empathy and post-traumatic growth (PTG) in school bullying victims. We screened 725 adolescents who had experienced school bullying as our final subjects out of the 2173 adolescents we surveyed over three periods (November 2019, 2020, and 2021). Controlling for gender, cross-lagged analysis revealed that both cognitive empathy at T1 and T2 predicted adolescents’ later PTG at T2 (γ = 0.096, *p* < 0.05) and T3 (γ = 0.085, *p* < 0.05), respectively, but the predictive effect across time points from PTG to cognitive empathy was not significant. The results delineated a specific directionality in the relation between cognitive empathy and PTG and suggested an important role of cognitive empathy in fostering school bullying victims’ later recovery and growth. These findings contribute to ongoing research into ways researchers and educators may help and support school bullying victims.

## 1. Introduction

Individuals who frequently endure aggressive behavior intended to cause harm—whether physically, psychologically, or socially—are identified as victims of bullying [1]. Research by Einarsen and his colleagues [2,3] presented the perspective that bullying can be equated to experiencing a traumatic event. A synthesis of numerous studies, both domestic and international, indicated that the prevalence of school bullying falls within a range of 20% to 33% [4]. Currently, school bullying has emerged as a chronic social issue, profoundly impacting the well-being and mental health of young individuals. This has led to heightened awareness and concern among nations globally [5].

Bullying victimization is increasingly recognized as a form of interpersonal violence that can be akin to a traumatic event [6]. Victims of bullying are susceptible to a spectrum of adverse outcomes, affecting them in both the short term and in the long term [7]. Nonetheless, a hopeful phenomenon was observed where, through personal resilience and external support [8], some individuals underwent a form of positive psychological transformation referred to as post-traumatic growth (PTG). This concept, introduced by Tedeschi and Calhoun [9], describes the beneficial psychological changes individuals experience as they navigate and overcome significant life crises. PTG encompasses three primary dimensions: transformations in self-perception, shifts in the nature of interpersonal relationships, and a re-evaluation of life’s priorities [10]. The journey toward PTG is rich in cognitive and emotional processing. Recent research endeavors have embarked on unraveling the intricate interplay between post-traumatic growth and empathy, hinting at a potential interconnectedness between these constructs [11,12].

Cognitive empathy is the capability to grasp the emotional state of another individual [13]. As outlined by Davis [14,15], cognitive empathy sits at the heart of empathetic understanding, focusing on the cognitive aspect of stepping into someone else’s shoes to view the world from their perspective. This form of empathy involves interpreting others’ experiences in a manner that facilitates effective social negotiation and interaction [16]. It plays a critical role in fostering healthy interpersonal connections and securing a more robust network of social support. Recognized as a valuable socio-emotional skill, cognitive empathy involves top–down processing [15,17,18]. It is mirrored in the ability to identify and comprehend the emotions and viewpoints of others, a competency crucial for navigating and adapting to the ever-evolving social landscape [19].

Post-traumatic growth (PTG) and cognitive empathy are increasingly recognized as vital components of positive psychology. A substantial body of research has explored the linkage between these two factors, identifying post-traumatic growth as a phenomenon deeply intertwined with extensive cognitive and emotional processing [8]. Further investigations have uncovered a significant positive correlation between empathy and post-traumatic growth. For instance, Wang’s research indicated a close connection between the level of PTG in individuals who have experienced trauma and their capacity for empathy [20]. However, it is noteworthy that the cognitive aspect of empathy has often been overlooked in research focusing on the dynamics between empathy and trauma derived from bullying [21].

According to de Waal and Preston’s [22] Russian-doll model, empathy is structured in hierarchies, layering from the inside out, with cognitive empathy positioned at the outermost layer and emotional resonance at its core. Research by Shamay-Tsoory et al. [23] found that emotional empathy and cognitive empathy exhibited a dual separation, both behaviorally and anatomically. Moreover, cognitive empathy typically emerges during childhood and adolescence, a developmental stage when individuals begin to adeptly assume the perspectives of others [24,25,26,27]. This development highlights that, particularly for adolescents, the advancement of cognitive empathy takes precedence over emotional empathy in importance [28]. Given these insights, our study opts to disentangle cognitive empathy from its emotional counterpart, investigating it as an independent variable.

Post-traumatic growth (PTG) and cognitive empathy share a complex, bidirectional relations. On the one hand, post-traumatic growth appears to unlock an individual’s latent cognitive empathy [11], while on the other, cognitive empathy may act as a catalyst for an individual’s post-traumatic growth [12].

Evidence suggests that cognitive empathy serves as a predictor for post-traumatic growth. Zhou et al. [12] demonstrated that the activation of cognitive empathy enabled individuals to adopt broader perspectives, facilitating the re-evaluation of trauma-related events and fostering personal growth. Similarly, An et al. [29] found that empathetic individuals, being more open to external influences, underwent stress and negative emotions that prompted reflection on traumatic events, thus aiding in post-traumatic growth. Calhoun and Tedeschi [30] highlighted the significance of empathy among people as a potential source of post-traumatic growth for individuals experiencing trauma. The longitudinal study conducted by Wang and Wu [20] further confirmed that empathy can significantly enhance subsequent post-traumatic growth. Notably, cognitive empathy, a crucial element as outlined by Shamay-Tsoory et al. [23], played a pivotal and indispensable role in this process. Moreover, secondary post-traumatic growth studies hinted that cognitive empathy may be more conducive to individual growth than affective empathy [31,32]. Furthermore, pertinent theories endorse the contention that cognitive empathy serves as a contributor to post-traumatic growth. Drawing from the interpersonal model of stress and coping, when stressful occurrences endanger the well-being of a group, effective coping mechanisms encompass not merely problem-solving and the regulation of stress-induced negative emotions, but also the preservation of harmonious interpersonal relationships. Empathy emerges as a prototypical interpersonal-oriented coping strategy [33], with cognitive empathy occupying a pivotal role in interpersonal exchanges [34]. Additionally, the cognitive processing theory of trauma posits that cognitive factors are crucial for post-traumatic growth [35], suggesting a significant impact of cognitive empathy on positive growth outcomes. Together, these research findings imply the important role of cognitive empathy for post-traumatic growth.

Conversely, research also indicates that post-traumatic growth could predict cognitive empathy. Growth stemming from one’s connection to vulnerability may enhance latent empathy, subsequently stimulating cognitive empathy [11]. Earlier studies suggested potential pathways where growth may lead to an enhancement in cognitive empathy [36,37,38]. The theory of suffering-based altruism [37] posits cognitive empathy as a potential outcome of growth, with psychological changes from trauma recovery enhancing cognitive empathy. Lifelong developmental models of empathy [39,40] and recent meta-analyses [28] further underscore the developmental trajectory of empathy, emphasizing that cognitive empathy, which evolves and strengthens over time, may be fostered by post-traumatic growth.

Yet, some research, such as that of Elam and Taku [41], pointed towards a non-significant relations between PTG and empathy, although it did not specifically dissect the relations between cognitive empathy and PTG. The relations between cognitive empathy and post-traumatic growth remains intricate, likely influenced by variables such as study populations, traumatic experiences, measurement timelines, and tools. Additionally, past relevant research paid less attention to adolescent victims of bullying [42]. Furthermore, a notable limitation in the existing literature is the reliance on cross-sectional designs, which hampers identifying definitive relationships and exploring causality.

This study, grounded in the interpersonal model of stress and coping and theories of suffering altruism, employs a longitudinal design with a cross-lagged approach. Our aim is to elucidate the relations and directionality between post-traumatic growth and cognitive empathy in the context of school bullying trauma, contributing valuable insights to empirical research in this domain.

## 2. Materials and Methods

### 2.1. Participants

In this investigation, we surveyed 2173 adolescents across Zhejiang Province, comprising 1384 junior high school students and 789 high school students. Following the guidelines proposed by Xie et al. [43], a participant was considered to have experienced bullying if they reported encountering such incidents “once or twice a month” or more often on any item of the Delaware Bullying Victimization Scale (student version). This criterion implicates that the individual has undergone bullying within the specific domain addressed by the question, thereby marking them as a victim of bullying. Applying this definition, we identified 802 out of the 2173 adolescents as having been bullied at the first time point. For this research, the focus was placed on these 802 adolescents who had encountered bullying. They were tracked and assessed at three distinct intervals: November 2019 (T1), November 2020 (T2), and November 2021 (T3). Due to various reasons such as school transfers and illnesses, some participants were unable to complete all three surveys. To ensure the reliability of the results, we retained subjects who had participated in at least two surveys, resulting in a sample size of 725 participants at the first time point. Within this group, there were 430 (59.3%) male students, 294 (40.6%) female students, and 1 individual who did not specify their gender. The average age of the participants was 14.44 years (SD = 1.40) at the first time point, with ages ranging from 10 to 18 years.

### 2.2. Measures

#### 2.2.1. Bullying Victimization

In this study, the Delaware Bullying Victimization Scale (student version) revised by Xie et al. [44] was used to assess adolescents’ school bullying victimization. The scale has 17 question items divided into four dimensions: verbal, physical, social/relational, and cyberbullying victimization, with item 13 as a screening item not counted in the data analysis. The items on the scale are scored on a six-point Likert scale, where 0 means “never,” 1 means “occasionally”, 2 means “once or twice a month,” 3 means “once a week”, 4 means “many times a week”, and 5 means “every day”. Higher scores indicate more severe bullying. Cronbach’s alpha coefficients for this scale in this study ranged from 0.902 to 0.961.

#### 2.2.2. Post-Traumatic Growth

In this study, the Post-Traumatic Growth Questionnaire (PTGQ) developed by Tedeschi and Calhoun [10] and revised by Zhou [45] was used. The questionnaire was designed to assess in what ways individuals changed significantly after experiencing bullying in school. The revised questionnaire consists of 22 questions, including three dimensions: changes in self-awareness, changes in interpersonal experiences, and changes in life values. The corresponding item numbers are 9, 7, and 6, respectively. The questionnaire is assessed on a 6-point scale, where 0 means “no change” and 5 means “very big change”. The higher the score on the scale, the stronger the PTG, i.e., the more growth. Cronbach’s alpha coefficients for this scale in this study ranged from 0.969 to 0.987.

#### 2.2.3. Cognitive Empathy

In this study, a five-item version of the perspective-taking dimension of the Interpersonal Reactivity Index [14,15] was used to measure cognitive empathy. The scale has 5 question items, and a sample item included, ‘‘When my friends are having a disagreement or an argument, I try to listen to everybody before I decide who is right.’’ Each item is scored on a five-point Likert scale (1 = completely inconsistent, 5 = completely consistent). Cronbach’s alpha coefficients for cognitive empathy in this study ranged from 0.841 to 0.914.

### 2.3. Procedure and Data Analysis

In this research study, several graduate students specializing in psychology were meticulously trained to serve as examiners. This comprehensive training covered the test’s content, administration requirements, procedural details, and essential precautions to be observed. Prior to test administration, the schools and teachers facilitated introductory sessions for parents and students to ensure a thorough understanding of the test’s objectives and methodology. The testing was uniformly conducted across all classes, with students completing the questionnaires under the supervision of an invigilator who subsequently collected them immediately. This protocol was consistently applied across all three testing sessions. At the end of the survey, all participants were informed that psychological/counseling services were available from school psychologists or teachers if needed.

Data analysis was performed using SPSS 26.0, employing techniques for descriptive statistics, correlation analysis, and testing for common method bias. Furthermore, Mplus 8.3 software was utilized to develop a structural equation model for cross-lagged analysis. Model estimation was carried out using robust maximum likelihood estimation (MLR), while the full information maximum likelihood estimation (FIML) approach was adopted to handle missing data, following the methodology proposed by Muthén and Muthén [46].

### 2.4. Testing for Common Method Bias

To address the potential for common method bias in this study, we applied the Harman single-factor test. This analysis revealed twelve factors with eigenvalues exceeding 1 across the three time-points of measurement. Specifically, the proportion of variance explained by the first factor, both before and after rotation, was 28.87% and 16.67%, respectively. These findings suggest that common method bias does not pose a significant concern in this study, allowing for a more confident interpretation of the results.

Subsequently, we employed descriptive statistics and correlation analyses to explore the relationships between levels of variables and the variables themselves. Furthermore, cross-lagged regression analyses were conducted to investigate the dynamic relations between cognitive empathy and post-traumatic growth among school bullying victims.

## 3. Results

### 3.1. Correlations between Post-Traumatic Growth and Cognitive Empathy

Table 1 displays the mean values, standard deviations, and Pearson correlation coefficients for the scores of post-traumatic growth and cognitive empathy among school bullying victims over a three-year period. The analysis revealed a positive correlation between the post-traumatic growth scores and cognitive empathy scores of school bullying victims at each respective time point, with all correlations being significant (rs ranging from 0.316 to 0.376, ps < 0.001). Furthermore, a significant positive correlation persisted over the course of the three years for both post-traumatic growth (rs ranging from 0.26 to 0.40, ps < 0.001) and cognitive empathy (rs ranging from 0.22 to 0.41, ps < 0.001) among the victims. Gender showed a significant correlation with both post-traumatic growth and cognitive empathy in school bullying victims, but age did not. As such, gender was incorporated as a control variable in subsequent cross-lagged modeling analyses to further investigate these relationships.

### 3.2. Cross-Lagged Analysis of Post-Traumatic Growth and Cognitive Empathy

To ascertain the dynamic interplay between post-traumatic growth and cognitive empathy among school bullying victims, we proceeded with cross-lagged analyses that necessitated verifying the measurement invariance of these constructs over the three assessment points. We established and examined models for configural invariance, weak invariance, and strong invariance for the measures of post-traumatic growth and cognitive empathy among school bullying victims, with model fits detailed in Table 2. 

A comparison was made between the configural invariance model (serving as the baseline model) against the weak invariance and strong invariance models. Although the chi-square test results indicated significant differences between the models, it is essential to note that the chi-square value is susceptible to sample size, rendering it a somewhat unstable indicator for model evaluation. Recognizing this limitation, it is prudent to consider variations in other fit indices when comparing models. In line with Cheung and Rensvold’s [47] recommendation, we employed the ΔCFI for model comparison, since it remains unaffected by model parameters and sample size. The criterion for accepting measurement invariance, as suggested by these authors, is a ΔCFI of 0.01 or less. By examining the discrepancies across multiple fit metrics, we determined that both post-traumatic growth and cognitive empathy among school bullying victims exhibited sufficient configural and weak invariance over time. This finding paved the way for conducting in-depth cross-lagged analyses to further explore the relations between these variables. 

**Figure 1:**
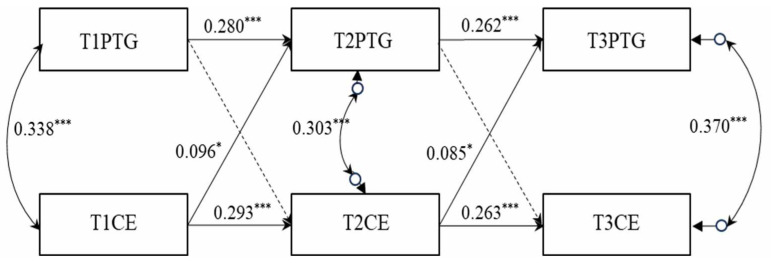
Cross-lagged analysis of post-traumatic growth and cognitive empathy. Note: *** denotes *p* < 0.001; * denotes *p* < 0.05; PTG = post-traumatic growth, CE = cognitive empathy. (In the model, solid lines indicate significant standardized path coefficients, while dashed lines represent non-significant path coefficients. For clarity, path coefficients related to control variables have been omitted from the visual representation).

Structural equation modeling was employed to explore the cross-lagged relations between post-traumatic growth and cognitive empathy among school bullying victims. In this model, gender served as a control variable, influencing both post-traumatic growth and cognitive empathy across the three time points. The results of this model are depicted in Figure 1, showcasing satisfactory model fits: Satorra–Bentler Scaled Chi-square (SBχ^2^) (8) = 13.507, *p* > 0.05; Comparative Fit Index (CFI) = 0.982; Tucker–Lewis Index (TLI) = 0.953; Root Mean Square Error of Approximation (RMSEA) = 0.031 (90% CI [0.000, 0.058]); Standardized Root Mean Square Residual (SRMR) = 0.039. To simplify model presentation, path coefficients for the control variable—gender—are not included in Figure 1. 

As illustrated in Figure 1, both post-traumatic growth and cognitive empathy demonstrated moderate stability over the course of the three-year observation, with autoregressive path coefficients ranging from 0.26 to 0.29 (ps < 0.001). When controlling for autoregressive effects and within-time correlations between post-traumatic growth and cognitive empathy, cognitive empathy at the first time point (T1) was found to significantly and positively predict post-traumatic growth at the second time point (T2) (γ = 0.096, *p* < 0.05). Similarly, cognitive empathy measured at the second time point (T2) significantly and positively predicted post-traumatic growth at the third time point (T3) (γ = 0.085, *p* < 0.05). Contrarily, post-traumatic growth at T1 did not significantly predict cognitive empathy at T2 (γ = −0.002, *p* = 0.948), nor did post-traumatic growth at T2 significantly predict cognitive empathy at T3 (γ = −0.002, *p* =.0.948). This analysis therefore highlighted a temporal association where cognitive empathy precedes and positively influences the development of post-traumatic growth in school bullying victims over time.

## 4. Discussion

This research study utilized a cross-lagged design across three time points to delve into the dynamic interplay between post-traumatic growth and cognitive empathy among adolescent victims of bullying. The outcomes indicate that pre-existing cognitive empathy in victims can positively influence subsequent levels of post-traumatic growth. However, the emergence of post-traumatic growth does not appear to reciprocally amplify cognitive empathy. These findings partially corroborate the interpersonal modeling theory of stress and coping, as well as the cognitive processing theory related to post-traumatic growth [35,48], aligning with the trajectories observed in longitudinal research [20].

### 4.1. The Role of Cognitive Empathy in Post-Traumatic Growth

This study uncovered that cognitive empathy in adolescents who have experienced bullying significantly fosters post-traumatic growth one year following the initial trauma. This finding broadens the longitudinal insight previously established by other researchers [49], offering a novel lens through which we can view the psychological recuperation of school bullying victims. This observation is supported by similar findings across various studies [12,20,50,51,52]. 

Cognitive empathy, or the capacity to understand the perspectives and emotions of others, is identified as a crucial socio-emotional skill [53], vital for sustaining healthy social relationships [54]. This skill enables individuals to recognize and respond to the needs of others effectively. Among victims of bullying, those with heightened cognitive empathy exhibit better interpersonal-focused coping mechanisms when confronted with trauma. These strategies facilitate their recovery and psychological development. This aligns with the anticipations made by the interpersonal modeling theory of stress and coping [48], which elucidates how people can more efficaciously manage stressful situations. Additionally, the cognitive processing theory of trauma [35] suggests that recovery from trauma necessitates the reintegration of disrupted beliefs and disconcerting information emanating from the traumatic event. The cognitive dimension of empathy allows individuals to transcend self-centered perspectives and objectively view and understand the event from others’ viewpoints, fostering recovery and post-traumatic progression. For bullying victims, heightened cognitive empathy can also engender a more reconciliatory stance towards perpetrators [55], transitioning from animosity towards others to self-improvement, enhancing adaptation to their environment, and fostering post-traumatic growth.

In addition, individuals exhibiting cognitive empathy are likely to adopt others’ perspectives on issues [56], which strengthens personal connections and facilitates receiving more support [57]. This not only aids in effectively mitigating the trauma’s adverse impacts but also enables victims to experience positive changes in their interpersonal relationships, further encouraging post-traumatic growth.

In summary, this result highlighted the significant influence of cognitive empathy on the psychological recovery and growth journey of school bullying victims. Enhancing cognitive empathy could potentially arm victims with more effective tools for coping with their trauma and achieving psychological development. These insights contribute to a deeper understanding of the psychological evolution of school bullying victims post trauma, offering valuable guidance for psychologists and educators in recognizing potential psychological outcomes and formulating suitable support strategies for victims. Moreover, by exploring post-traumatic growth among school bullying victims, this research introduces fresh perspectives and insights into the field, emphasizing that post-traumatic growth extends beyond catastrophic events to include daily life challenges such as bullying.

### 4.2. The Role of Post-Traumatic Growth in Cognitive Empathy

Contrary to initial predictions, this study revealed that post-traumatic growth observed in adolescents who had experienced bullying did not serve as a predictor for cognitive empathy in the subsequent year. According to Tedeschi and Calhoun [10], post-traumatic growth represents a positive change across three domains—self-perception, interpersonal relationships, and life’s perceived value—following a traumatic event. Such transformation is a gradual process, and Yan et al. [28] asserted that the period from mid-childhood to early adulthood is primarily characterized by the development of cognitive empathy. However, the three-year duration of our study may not have been adequate to capture post-traumatic growth’s potential to enhance cognitive empathy.

Moreover, the development of cognitive empathy in victims of bullying could be influenced by various factors, including previous bullying encounters. While our research focused on individuals who have suffered bullying, it failed to distinguish among different forms of bullying victimization. A recent meta-analysis by Meng et al. [58] proposed that the nature of the traumatic experience mediated the relations between trauma and empathy. Furthermore, the experience of post-traumatic growth and its influence on cognitive empathy might vary among individuals. For instance, Yan et al. [59] observed that women exhibited significantly higher levels of empathy compared to men. This is consistent with the results of this study, which found that women had higher levels of cognitive empathy at the T2 time point compared to men. Consequently, gender could play a certain role in mediating the relations between post-traumatic growth and cognitive empathy. But gender differences were not the main purpose of this study and gender was shown to be associated with cognitive empathy and post-traumatic growth only at the T2 time point. Therefore, while this study controlled for gender, it did not break down study results by men and women. This might have hidden specific gender-related dynamics in the interplay between post-traumatic growth and cognitive empathy. In future research, a deliberate classification of subjects by gender for separate analyses could offer deeper insights.

## 5. Implications and Limitations

The present study revealed that the cognitive empathy of victims of school bullying could predict their subsequent post-traumatic growth. This finding suggests that victims with higher levels of cognitive empathy are more likely to achieve positive psychological transformation and growth after experiencing bullying. These findings will enable a more comprehensive exploration of the evolving interplay between post-traumatic growth and cognitive empathy among bullying victims. Such efforts are anticipated to foster an understanding of the dynamics governing the relation between post-traumatic growth and cognitive empathy among this demographic and to refine interventions for enhancing bullying victims’ post-traumatic growth by improving their cognitive empathy levels. Cognitive empathy training can be added to the intervention program for the bullied person; for example, cognitive empathy can be enhanced through methods and tools such as role-playing, emotional transposition, and empathy training games [60,61,62].

The current study also has some limitations. Firstly, while employing a longitudinal study design, the reliance on questionnaires for all three data collection points may have introduced susceptibility to social desirability biases and potential common method biases. Future studies are encouraged to incorporate methods such as interviews and observations to enhance study validity. Secondly, the current study was conducted during the COVID-19 pandemic, so some psychological responses may have been influenced to some extent by pandemic exposure. Future studies should control for COVID-19 pandemic exposure as a covariate when assessing psychological responses in the context of school bullying. Thirdly, the examination of the link between post-traumatic growth and cognitive empathy among bullying victims was confined to middle school students, predominantly from a specific city in Zhejiang Province, China. This limitation restricts the broader applicability of this study’s findings. Future research endeavors will aim to broaden the sampling scope, employ diverse methodologies, and integrate measurements at various intervals. 

## Figures and Tables

**Table 1:** Correlation analysis.

	M	SD	Sex	age	T1PTG	T2PTG	T3PTG	T1CE	T2CE	T3CE
sex	—	—	1							
age	14.44	1.4	0.014	1						
T1PTG	54.6	30.8	−0.052	−0.012	1					
T2PTG	48.6	30.2	−0.115 *	−0.054	0.263 ***	1				
T3PTG	46.7	33.3	0.014	−0.078	0.266 ***	0.395 ***	1			
T1CE	17.4	4.9	0.008	0.03	0.334 ***	0.182 ***	0.077	1		
T2CE	17.2	4.4	0.106 *	−0.038	0.073 #	0.316 ***	0.134 *	0.270 ***	1	
T3CE	16.6	4.5	−0.039	−0.005	0.1 #	0.095	0.376 ***	0.126 *	0.308 ***	1

Note: *** denotes *p* < 0.001; * denotes *p* < 0.05; # denotes borderline significant; PTG = post-traumatic growth, CE = cognitive empathy; gender coded as male = 0, female = 1.

**Table 2:** Measurement invariance test.

		χ^2^	df	CFI	TLI	SRMR	RMSEA (90%CI)	ΔCFI	ΔRMSEA
PTG	Configural invariance	4053.911	1943	0.924	0.916	0.038	0.039 [0.037, 0.040]	—	—
	Weak invariance	4133.524	1981	0.923	0.916	0.039	0.039 [0.037, 0.040]	−0.001	0
	Strong invariance	4217.308	2019	0.921	0.916	0.041	0.039 [ 0.037, 0.040]	−0.002	0
CE	Configural invariance	93.428	69	0.991	0.987	0.037	0.022 [0.008, 0.033]	—	—
	Weak invariance	103.641	77	0.99	0.987	0.043	0.022 [0.009, 0.032]	−0.001	0
	Strong invariance	110.135	85	0.991	0.989	0.043	0.020 [0.006, 0.030]	0.001	0.002

Note: PTG = post-traumatic growth, CE = cognitive empathy.

## Data Availability

The data that support the findings of this study are available from the corresponding author or the first author upon reasonable request.
